# How Do Dogs Behave When Presented with Situations of Different Emotional Valences?

**DOI:** 10.3390/ani13061027

**Published:** 2023-03-11

**Authors:** Paulo Souza, Kun Guo, Daniel S. Mills, Briseida Resende, Natalia Albuquerque

**Affiliations:** 1 Institute of Biosciences, University of São Paulo, São Paulo 05508-090, Brazil; 2 School of Psychology, University of Lincoln, Lincoln LN6 7TS, UK; kguo@lincoln.ac.uk; 3 Department of Life Sciences, University of Lincoln, Lincoln LN6 7TS, UK; dmills@lincoln.ac.uk; 4 Institute of Psychology, University of São Paulo, São Paulo 05508-030, Brazil; briseida@usp.br

**Keywords:** behaviour, *Canis familiaris*, cognition, emotion, social cognition, valence

## Abstract

**Simple Summary:**

Understanding how dogs behave in different situations is an important question in the dog–human relationship. In fact, it is not yet fully understood how their behaviours might be linked to their emotional state, which generates obstacles to communication between species. The aim of this study was to investigate how different emotions (positive, negative and neutral) affect dogs’ behaviour by using an experimental setup from previous research. In the current study, dogs were exposed to a specific emotional expression from one of two actors in a room where there was food available in two ways: directly or indirectly (dogs needed the human for help). Our results show that, in positive conditions, dogs tend to explore the environment more than in negative ones, and there are two behaviours that arise that might indicate their search for information and possibly a positive emotional reaction in this situation: their tail raised between 90° and 180° and physical contact during sniffing. This study should encourage more research on the link between behaviour and emotional states in dogs.

**Abstract:**

Dogs are good models for studying behaviour and cognition as they have complex social capabilities. In the current study, we observed how human emotional valences (positive, neutral and negative) affected aspects of dogs’ behaviour. We expected that dogs would exhibit more approaching behaviours in the positive condition and more signs of avoidance in the negative one. We analysed videos of 70 adult pet dogs of various breeds taken from an experiment in which one of two actors expressed an emotion and dogs could freely explore the environment for 30 s. Our results show that dogs exhibit differential behaviour when presented with different emotional valences. Two behaviours arose that might be linked to a reciprocal positive emotional state in dogs: tail raised between 90° and 180° and physical contact during sniffing. These behaviours are associated with an active search for information. In the positive conditions, dogs were more willing to explore the social environment and gather information from the actors.

## 1. Introduction

The study of emotional and mental states in animals is hampered by a language barrier. Therefore, it is necessary to make use of inferential techniques, such as investigations focused on emotionally relevant behaviours (e.g., body postures, tail wagging) to better understand how animals express what they are feeling. Dogs possess a wide repertoire of signals to interact with the environment and communicate their responses to different situations [1]. They can express themselves through facial expressions [2,3,4], which are sensitive to human attentional state [5], body posture [6,7], tail wagging [8] and vocalisations [9,10]. They are able to do so in distinctive ways depending on the emotional salience of a stimulus (positive or negative) that they are responding to. For instance, it has been shown that there are acoustic differences in the quality of vocalisations (i.e., rhythmicity, tonality and frequency) emitted by individuals in different situations, such as disturbance, isolation or play (e.g., [10,11]). On the other hand, dogs have the ability to discriminate [12], categorise [13] and recognise [14] emotional expressions from humans and other dogs [15]. They differentiate positive from negative emotions, as has been demonstrated in experiments where subjects were shown facial expressions of different valences associated with vocalisations: dogs not only matched the emotional content of the stimuli [14] but also reacted with differential behavioural responses, such as exhibiting mouth-licking more often when observing a negative human facial expression than when seeing a positive one [16]. Other studies involving emotionally salient cues, such as body posture and visual contact, have demonstrated dogs’ discrimination between a friendly and threatening unfamiliar person by showing different reactions to the associated approaches (e.g., [17]). Our team’s recent work [18] has revealed that dogs can infer emotional states of humans since, when they are exposed to actors exhibiting facial expressions of different emotional valences, they can use this information in a functional way to make decisions [18]. This requires cognitive abilities thought, until recently, to be exclusive to primates [18]. The latter experiment consisted of two actors interacting silently while exchanging a neutral object and reacting in three possible manners: with a positive (happy), negative (angry) or neutral facial expression. This was followed by a task in which the dog had to access a bowl with food. Access could be direct (i.e., dogs could reach the food by themselves) or indirect (i.e., dogs needed the actors to reach the baited bowl for them). To investigate whether dogs would use the available emotional information differentially depending on the condition with which they were presented, we looked at approaching behaviour, choices, gazing, body orientation, looking, position in the room and sniffing. It was found that the display of behaviours, such as gazing more at the upper half of the human’s body, relied on the accessibility of the food and on the emotional information provided as the dogs gazed at the upper half of the positive actor’s body for more time in the positive condition and at the upper half of the neutral actor’s body for more time in the negative condition. However, many aspects of behaviour, such as responses of agitation, willingness to explore the environment and interest associated with each condition, were not evaluated. The aim of the current study was to use the videos generated by the previous work [18] to test the hypothesis that dogs show differential behaviours in response to different emotionally charged situations. We did so by examining more closely how dogs behave in situations involving humans expressing different emotional valences (positive, negative and neutral). For this intent, we created a new ethogram that contained measurable behavioural categories and behaviours that are informative about dogs’ exploratory behaviour and how they perceive the environment they are in based on the emotional valence of the situation. We predicted that certain behaviours would be prevalent depending on the emotional condition. For instance, behaviours associated with openness, such as approaching, sniffing or jumping on, would be more likely to occur in the positive context, whereas showing no interest would occur more often in the neutral condition and behaviours related to avoidance, such as hiding behind the owner, would appear more in the negative situation.

## 2. Materials and Methods

### 2.1. Subjects

For this study, we used 75 dogs (Table S1) from the sample of Albuquerque and colleagues (2021) [18], which had been randomised for emotional valence, role of the actor (giver or receiver of the object) and side. Five of the subjects had to be excluded due to camera limitations. Our sample consisted of 45 female and 25 male healthy domestic pet dogs *(Canis familiaris)* of various breeds. We used a between-subjects study design, meaning that each subject was tested once. The dogs were used to living with humans and to interacting with new people and environments. To be included in the analysis, the dog must have looked at both actors during the demonstrations for at least 5 s, have not exhibited any signs of stress during the baseline observation phase and the test must have occurred without interference of external noises or interaction of the owner towards the dog during the response phase. 

Ethical approval was granted by the Animal Ethics Committee of the Instituto de Psicologia of Universidade de São Paulo, Brazil, and all procedures complied with the ethical guidance for use of animals by the International Society for Applied Ethology.

### 2.2. Procedures

**Figure 1:**
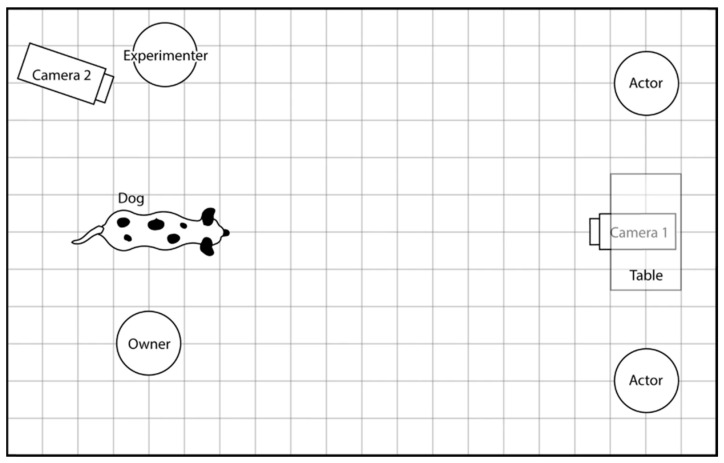
Scheme of the experimental environment and the position for each element (images from Albuquerque et al. and taken by themselves in 2021).

Subjects were tested in a room with two chairs and a table against the wall with two bowls with food [18]. Before the test, dogs underwent a demonstration phase where they observed two actors (see details below) interacting with each other. Three black discs were used as neutral tokens of exchange between the actors (they were novel to all the dogs and selected to have no meaning) in order to capture the dogs’ attention and create an interaction that was neutral but at the same time could lead to an emotional response. As this is a naturalistic experiment, we aimed at providing dogs with the most natural experience possible, hence an exchange of disks between the two demonstrators, where one person gives “something” and the other receives “something”. This created a social dynamic that is common for dogs and that can elicit emotional exchange of information. All dogs saw the demonstration before taking part in the test. The food (dried meat treats) was used to incentivise the dogs to approach this test area as they were on the other side of the room beside their owners. The dogs were not food-deprived, but the owners were instructed to not feed the dogs two hours before the experiment. A camera was placed below the table to provide a frontal angle of the dogs. The room had markings indicating where every element should be (i.e., owner, experimenter, actors, dog and table) so the positions did not vary between tests (Figure 1). On the other side of the room, there was another camera positioned in the corner. Between tests, the room was cleaned with water and disinfectant to avoid scent cues from other dogs. 

#### 2.2.1. Pre-Experiment 

Dogs were habituated to the environment before testing, which lasted between five and ten minutes. After this, the owner and the dog went to a separate area while the actors entered the experimental room and positioned themselves in front of the chairs. A helper also entered the room, put the food (dried meat treats) in the bowls and started the cameras. The owner and the dog were instructed then to return to the experimental room. Upon entering the experimental room, the owner was asked to go with the dog to the table and, ignoring the actors, present the two bowls to the dog until it showed interest. After the dog had seen and smelled the food, they returned the bowls to the table. The dogs did not get an opportunity to eat the food at this point. After that, both went to their designated marking at the other end of the room, as shown in the figure below, and stood there until the owner was given the command to release the dog from the leash. From that point on, nobody interacted with or responded to the dog in any way (see [18] for more details).

#### 2.2.2. Experimental Procedure

**Figure 2:**
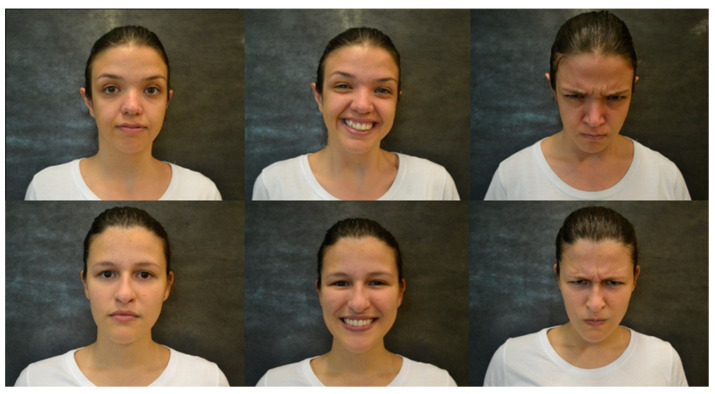
Examples of the facial expressions displayed by the actors. From left to right: neutral, positive (happiness) and negative (anger) (images from Albuquerque et al. and taken by themselves in 2021).

**Figure 3:**
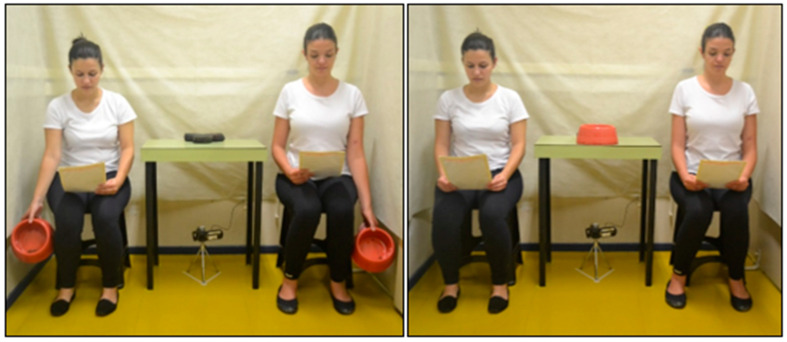
Different conditions of food availability. On the left, there is the direct scenario, and, on the right, there is the indirect (images from Albuquerque et al. and taken by themselves in 2021).

The experiment consisted of two phases. First, the observation phase, in which the dogs were presented with a social interaction between the two unfamiliar humans (actors). The actors were two Caucasian females of the same age trained beforehand. During all tests, they wore the same clothes, had their hair wrapped in the same way, wore no makeup, jewellery or perfume. In the interaction, the actors had a giver–receiver dynamic with the three black discs. At the start, both were quiet, still and neutral in facial and body expressions, standing beside the table with the two bowls with food and the three discs. One of the actors would then pick up a disc, always displaying an emotionally neutral facial expression, and deliver it to the other actor, who, upon receiving it, would turn their head and express either a positive (happiness), negative (anger) or neutral facial expression (Figure 2) and then return the disc to the table. This process happened three times, each with different discs in order to make sure the dog had a chance to observe the exchange for at least 5 s. The two actors would then pick up a piece of paper and sit on their respective chairs looking down and remaining with neutral facial and body expressions (see video S1). The owner then received a cue to release the dog from the leash and, upon doing so, the dog was free for 30 s to explore the environment and interact with its elements (Videos S2 and S3). There were two possible scenarios for accessing food: in the direct condition, each actor held a bowl with one hand, giving direct access to the dog to reach it; in the indirect condition, both bowls were on the table (Figure 3), so the dog would need to interact with one of the demonstrators to access either bowl. Which actor gave and received the discs, the facial expression displayed and the availability of the bowls was randomised between subjects. Both phases were recorded with two cameras, and images of the response phase were put together side by side to make one single file that enabled coding from both angles.

### 2.3. Coding

We used the ethogram from Table 1 to describe the dogs’ behaviour when facing situations of different emotional valence. This ethogram was designed specifically for this study based on observations of video recordings and some categories previously considered [18]. 

The ethogram was then turned into a coding sheet for the software Solomon Coder Beta v. 19.08.02, which was used to code all the videos. The coding was blind as the coder did not know which emotional condition was being shown in the videos. A second person double-coded 25% of the videos, and reliability tests (ICC) were run between both coders, yielding high values (see Table S2 in Supplementary Materials for details). ICC was completed for multiple raters using two-way mixed effects model and absolute agreement. Body direction towards the experimenter and head towards the table did not present similar results, showing low values. Therefore, they were excluded from analysis. 

### 2.4. Data Analysis

For analysis, two main datasheets were generated, one with the duration of behavioural categories (body posture, body direction, head direction, tail position, tail movement (type), tail movement (presence), physical contact, sniffing, jumping on and reaction) and one with the frequency data (approach and course). The latter considered the first approach of the subject and whether the trajectory was direct (straight to demonstrators and table from the starting point) or indirect. For each category, there is a “non-codable” option, which resulted in a difference in the test time for each subject. To account for this difference, we used only the codable time as the basis for analysis, with durations converted to a proportion for each subject. To check if the emotional (test) conditions resulted in differences in the codable/non-codable time, we ran an ANOVA using the proportion of codable time as dependent variable. We found no significant differences.

First, we undertook a descriptive analysis of all variables within each category before undertaking multivariate or bivariate analyses depending on the number of related variables (behaviours) analysed. We used the Wald statistic to test the two factors (emotion and food availability). Residuals of the models were visually inspected for their distribution. IBM SPSS 25 was used for all the analyses. A 5% significance threshold was applied for interpretation of results, with no correction made for multiple testing, in line with the recommendations of Perneger [19]. For each category with the nature of the behavioural metric, associated variables and models used are described below. 

For **body posture** (continuous data), of the six possible behaviours, *stretching*, *avoidant*, *fearful*, *alarm* and *playful* were rare events, so they were not included in the analysis. This left *body posture neutral*, which was analysed as the dependent variable in a univariate generalised linear model with emotion and food condition as fixed factors. 

For **body direction** (continuous data), we used a multivariate general linear model including *owner*, *experimenter*, *demonstratorA*, *demonstratorB*, *table*, *bowl* and *out* as dependent variables and emotion and food condition as fixed factors. 

For **head direction** (continuous data), we used a multivariate general linear model including *owner*, *experimenter*, *demonstratorA*, *demonstratorB*, *table*, *bowl* and *out* as dependent variables and emotion and food condition as fixed factors. 

For **tail position** (continuous data), of the six possible behaviours, *raised-3*, *between legs* and *lowered* were rare events, so they were not included in the analysis. The remaining behaviours were tail position *raised-1*, *raised-2* and *relaxed*. These data were examined using three univariate generalised linear models, including each of the behaviours as a dependent variable in each model and emotion and food condition as fixed factors.

For **tail movement-type** (continuous data), from the three possible behaviours, *circular* and *linear vertical* were rare events, so they were not included in the analysis. This left tail movement *linear horizontal,* which was examined using a univariate generalised linear model, with emotion and food condition as fixed factors.

For **tail movement-presence** (continuous data), we used two univariate generalised linear models, including *moving* and *not moving* as dependent variables in each model and emotion and food condition as fixed factors.

For **physical contact** (continuous data), from the six possible behaviours, *licking*, *sitting on*, *leaning* and *touching* were rare events, so they were not included in the analysis. The remaining behaviours were *sniffing* and *jumping on*. To test these data, we used two univariate generalised linear models, including each of these behaviours as a dependent variable in each model and emotion and food condition as fixed factors. Because we found significant results regarding **sniffing** and **jumping on**, we ran additional descriptive analyses for these two behaviours and used the findings to better interpret the results from **physical contact**. 

For **reaction** (continuous data), from the three possible behaviours, *hiding behind owner* and *hiding behind experimenter* were rare events, so they were not included in the analysis, leaving only reaction of *no interest in the task*. To test these data, we used a univariate generalised linear model, including *no interest* as dependent variable in each model and emotion and food condition as fixed factors.

For **1st approach** (frequency data), there were five possible outcomes: *owner*, *experimenter*, *demonstratorA*, *demonstratorB* and *table*. For **course** (frequency data), there were two possible behavioural qualities: *indirect* course from the initial position to the demonstrators’ area and *direct* course to the demonstrators’ area. For both, we found more than 20% of cells having an expected count of less than five; therefore, we conducted descriptive analysis only. 

## 3. Results

Emotion and food condition showed no significant effects on **body posture**, **body direction**, **head direction**, **tail movement** and **reaction**.

**Figure 4:**
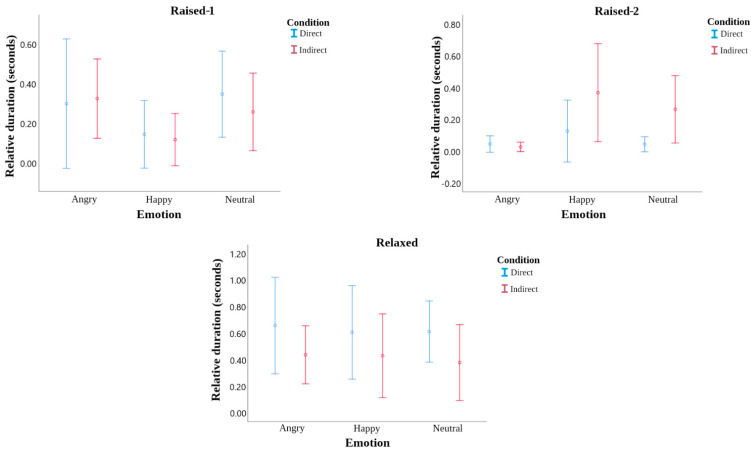
The relationship between the relative time (measured in seconds, *Y* axis) dogs spent with the tail in the raised-1 (tail forming an angle of 180° with the dog’s spine), raised-2 (tail forming an angle between 90° and 180° with the dog’s spine) and Relaxed positions in the direct and indirect food availability conditions (blue and red bars, respectively) and in the different emotional conditions of the test (*X* axis). Negative (angry) condition is shown on the left, neutral condition in the centre and positive (happy) condition on the right. Results show that subjects kept their tails at an angle between 90° and 180° in relation to their spine more in the positive condition and indirect condition, and they had their tail in the relaxed position longer in the direct condition than in the indirect.

For **tail position**, we found that emotion and food condition had a significant effect on tail position *raised-2* (tail raised in an angle between 90° and 180°), with tail position *raised-2* occurring more in the happy situation (mean ± se = 0.254 ± 0.05; X² = 12.639 gl = 2 *p* = 0.002) than in the neutral (0.148 ± 0.04) or negative (0.004 ± 0.04) conditions and more in the indirect (0.208 ± 0.03; X² = 6.219 gl = 1 *p* = 0.013) than the direct (0.063 ± 0.04) condition (see Figure 4 and Video S2 in Supplementary Materials). Moreover, food condition had an effect on tail position *relaxed* as it occurred significantly more in the direct condition (0.625 ± 0.07; X² = 4.345 gl = 1 *p* = 0.037) than in the indirect (0.414 ± 0.06). Neither emotion nor food condition had a significant effect on tail position *raised-1* (tail raised at 180°). 

For **physical contact**, we also found significant effects of emotion and food condition. Data showed that food condition and the interaction between emotion and food condition had a significant effect on *sniffing*, with the behaviour being exhibited for longer in the indirect (0.074 ± 0.01; X² = 3.846 gl = 1 *p* = 0.05) than in the direct condition (0.022 ± 0.02) and in the happy*indirect situation (0.165 ± 0.03; X² = 6.653 gl = 2 *p* = 0.036) (see Video S3 in Supplementary Materials). The descriptive analyses showed that dogs spent more time sniffing the demonstrators (*Demo A* = 0.031 ± 0.06; *Demo B* = 0.031 ± 0.09) than the experimenter (0.001 ± 0.01) or the owner (0.001 ± 0.01). The interaction between emotion and food condition was also significant for *jumping on* (X² = 7.415 gl = 2 *p* = 0.025), with the behaviour occurring more in the neutral indirect condition (0.083 ± 0.01). In this condition, dogs spent more time jumping on demonstrator A (0.011 ± 0.04) compared to demonstrator B (0.002 ± 0.01). Demonstrator A was always on the right side of the room. Further, we ran generalised linear models to see whether emotion and food condition had an effect on “moving the tail” and “not moving the tail”, but we found no significant results.

Finally, we ran descriptive analyses for **1st approach** and **course** and found that, in the happy situation, none of the dogs first approached the table in preference to the humans in the room. Moreover, we found that, out of the six dogs that did not move away from the owner during the entire test, five were in the negative emotional valence group. 

## 4. Discussion

We investigated the behaviours that dogs exhibit when presented with situations involving distinct human emotional valences in a setting where both emotion and food availability varied. We found that only tail positions *raised-2* (tail forming an angle between 90° and 180° with the dog’s spine) and *relaxed,* and physical contact (*sniffing* and *jumping on*) were affected by human emotional valence and food accessibility. It has been found [17] that dogs react differentially when approached by a stranger in a friendly or threatening way, which suggests that occurrence of some behaviours depends on the emotional valence being displayed. More recently [16], it was shown that dogs react to negative emotional displays (human faces with angry expression) with mouth-licking, which supports the suggestion that dogs are sensitive to human emotion and respond with different behaviours. 

Our current results suggest that, in general, dogs were seeking information in the room and that, in the positive valence situation, this was stronger. The previous work in the current experimental setting [18] showed that dogs considered the emotional information displayed by the two demonstrators (A and B, on the right and on the left) in problem-solving in order to decide with whom to interact. The latter study also found that the emotional information was more important for the subjects when the desired food was not reachable, that is, when the dogs could see but not get to the food by themselves and needed the humans in the room to help them. These findings, alongside our current results, point to dogs actively searching for emotional information during testing. *Sniffing* behaviour appears to be an example of that. 

*Sniffing* occurred longer in the indirect food availability condition, and the interaction with emotion was significant, showing that this behaviour happened more in the happy indirect condition. This would seem to indicate that *sniffing* may be involved in active acquisition of information. We hypothesise that, in a condition where the emotional valence is positive and there is a need to obtain information about the people involved in the task, it was safe to explore the environment, move away from the owner and approach the demonstrators. Use of olfactory perceptual modality is directly linked with information acquisition being used during problem-solving [20]. Sniffing is an important behaviour for detection and discrimination of odours that is modulated by sensory input during investigation, and its pattern may be used alongside trained alert responses to help determine detection [21]. Sniffing is used for odour investigation, during which asymmetries in nostril use can occur based on the emotional valence of the odour being investigated [22], and the information is encoded into their working memory [23]. In our study, sniffing occurred significantly more in positive situations, which would allow space for investigation. Moreover, means were higher (equally) for both demonstrators compared to the other humans in the room. When the situation was positive and there was indirect access to food, dogs spent more time sniffing the demonstrators, so sniffing might be an exploratory behaviour associated with acquisition of emotional information. In fact, studies show that the olfactory system plays an important role in detecting emotional cues of happiness and fear via human chemosignals [24]. 

Furthermore, we found that dogs’ tails were more often at the position *raised-2* in the indirect condition and in the happy emotion condition, meaning that the dogs’ tails stayed between 90° and 180° for longer when food was not reachable and when the demonstrators had interacted in a positive manner. This could be related to an alert exploration to obtain information and, since the information they received was that they were in a positively charged environment via the happy facial expression, there was space for moving through the room and approaching the humans in order to reach the food; there was no conspicuous sign of a negative outcome or response. Studies (e.g., [25]) suggest that tail up can be associated with a positive emotional state of an animal, which would be consistent with our findings regarding the positive valence situations. For the tail position *relaxed*, we found longer durations for the direct condition, which might be related to a lack of need for more information as the food was directly accessible; thus, there was less active exploration and the tail became relaxed. Tail movement (whether the tail was moving or not) did not show any significant relationships, which means the dogs moved their tails equally regardless of emotional context and food availability. An important aspect that must be taken into consideration is a potential effect of different breeds, which may vary in tail morphology. In our study, we have not discarded dogs because of the length/shape of the tail, and this should be further investigated. 

Another significant result was found for *jumping on* behaviour in the relation between condition and emotion. *Jumping on* occurred more in the neutral indirect condition, meaning it occurred for longer when there was no information available about the emotional state of the demonstrators, suggesting that it may be involved in more pronounced search for information, such as in a “search for help” situation. Little is known about the factors that affect “jumping on people” behaviour in dogs. However, jumping on is not arbitrary. According to previous findings [26], there are certain patterns involved in this behaviour. For instance, dogs discriminate between targeted people [27]. Moreover, jumping on people is affected by context and human body position [26]: there is possibly an effect of novelty and evidence to suggest that, when jumping on people, dogs may be trying to get close to the person’s face. In the current study, means were higher for demonstrator A, who was always on the right side of the table, regardless of identity or emotional state, meaning there was a side bias. A side bias could be related to lateralisation of the brain [28]; i.e., positive emotion may be processed more by the left side of the brain and, contralaterally, there could be a cognitive bias towards the right. Brain lateralisation is well documented in dogs, occurring in relation to tail wagging [8], visual exploration of faces and facial expressions [29,30], acoustic exploration of the environment [11], among other contexts. The side bias could also be due to the presence of the owner on the right side of the room, which might be a possibility since studies show that presence of a familiar human affects a dog in a positive way, causing, for example, hormonal changes, such as reduction in plasma cortisol [31]. Thus, having the owner on the right could have biased dogs towards the same side. 

For our analyses, body direction, head direction, tail movement and posture were not affected by food accessibility and emotional condition. One possible explanation would be that body language is related to emotion but perhaps not unique to a specific state, therefore not yielding significant results. Moreover, this lack of significance could mean that these behaviours were not as informative about the dogs’ response to emotional states as those considered above. For instance, other studies have shown that dogs’ focus is often related to their gaze [32], which may explain why body direction and head direction did not return significant results; i.e., these variables may not be sensitive enough for the purpose of this study. One important non-significant result is related to “No Interest”, one of the possible reactions to the experimental setting. “No Interest” was not affected by the two factors (emotion and food condition), which probably means that the dogs were interested enough in the task regardless of valence and may have engaged in testing equally. The relative durations for this behaviour varied from 0.04 to 0.9, with a low mean of 0.18 ± 0.26.

## 5. Conclusions

Our results show that dogs exhibit differential behaviour when presented with different human emotional valences and different food accessibility conditions. In the presence of a positive human emotional valence, dogs seem to have behaved more in an exploratory way, specifically when they needed the human to have access to food. Food accessibility and emotional condition played a significant factor in their willingness to explore the environment. Moreover, sniffing behaviour, jumping on people and a raised tail (specifically between 90° and 180°) appear to be linked to an active search for emotional information and seem to be positively related to positive emotional context. This study lays the groundwork for further investigations on behavioural responses potentially linked to emotional states and decision-making, which will help to better understand emotion perception in dogs and their relationship with people.

## Figures and Tables

**Table 1:** Ethogram with all the behaviours we looked at in this study.

Category	Behavioural Quality	Definition
Body Posture	Neutral	Relaxed head, back, hips and tail. The dog can be standing, sitting or lying down.
	Stretched	Stretched neck and body, hips can be raised or lowered, stretched front and/or back paws.
	Avoidant	Deviated head preventing visual contact, body directed away from the target, piloerection may occur, lowered tail or in between the legs. The dog can be standing, sitting or lying down.
	Fearful	Low head, shrunken body, lowered tail or in between the legs, retracted legs. The dog can be standing, sitting or lying down.
	Alarm	Raised head, erect body, stiff tail, piloerection may occur, stiffening of the musculature
	Playful (Play Bow)	Relaxed head, curved body, raised hips, raised and/or wagging tail, front paws flexed forward.
	Non-Codable	When the behaviour cannot be seen or distinguished.
Body Direction	Directed to owner	Body oriented towards the owner.
	Directed to experimenter	Body oriented towards the experimenter.
	Directed to demonstrator A	Body oriented towards demonstrator A.
	Directed to demonstrator B	Body oriented towards demonstrator B.
	Directed to table	Body oriented towards the table.
	Directed to bowl	Body oriented towards the bowl.
	Out	Body oriented in any direction besides the key elements of the experiment.
	Non-Codable	When the behaviour cannot be seen or distinguished.
Head Direction	Directed to owner	Head oriented towards the owner.
	Directed to experimenter	Head oriented towards the experimenter.
	Directed to demonstrator A	Head oriented towards demonstrator A.
	Directed to demonstrator B	Head oriented towards demonstrator B.
	Directed to table	Head oriented towards the table.
	Directed to bowl	Head oriented towards the bowl.
	Out	Head oriented in any direction besides the key elements of the experiment.
	Non-Codable	When the behaviour cannot be seen or distinguished.
Tail Position	Raised-1 (180)	Raised tail forming an angle of 180° in relation to the dog’s spine.
	Raised-2 (90)	Raised tail forming an angle between 90° and 180° in relation to the dog’s spine.
	Raised-3 (<90)	Raised tail forming an angle smaller than 90° in relation to the dog’s spine.
	Between the legs	Tail positioned in between the dog’s paws and towards the stomach.
	Lowered	Tail positioned down and close to the dog’s paws.
	Relaxed	Tail in a neutral position (i.e., without overt muscular engagement).
	Non-Codable	When the behaviour cannot be seen or distinguished.
Tail Movement (type)	Circular	Circular movement of the tail in relation to the dog’s hips.
	Linear horizontal	Linear movement of the tail in the horizontal direction.
	Linear vertical	Linear movement of the tail in the vertical direction.
	Non-Codable	When the behaviour cannot be seen or distinguished.
Tail Movement (presence)	Moving	When dog is moving its tail. Measured in duration and frequency.
	Not Moving	When the dog’s tail is not moving. Measured in duration and frequency.
	Non-Codable	When the dog’s tail cannot be seen.
Approaching	Owner	Entrance in the owner designated area.
	Experimenter	Entrance in the experimenter designated area.
	Demonstrator A	Entrance in the demonstrator A designated area.
	Demonstrator B	Entrance in the demonstrator B designated area.
	Table	Entrance in the table designated area.
	Non-Codable	Dog does not approach any of the key elements of the experiment.
Course	Direct	The route of the first approach to the demonstrators happened without deviations.
	Indirect	The route of the first approach to the demonstrators happened with deviations.
	Non-Codable	Dog does not approach the table or the demonstrators.
Physical Contact	Sniffing *	Active contact of the dog’s nose with any of the people in the experimental environment.
	Jumping on *	Active elevation of the dog’s entire body, touching any of the people in the experimental environment.
	Licking	Active contact of the dog’s tongue with any of the people in the experimental environment.
	Sitting on	Active contact of the posterior part of the dog over the foot/feet of any of the people in the experimental environment.
	Touching (passive)	Passive physical contact between the dog’s body and any of the people in the experimental environment.
	Touching (paws)	Active use of the dog’s paws to establish physical contact with any of the people in the experimental environment.
	Non-Codable	When the behaviour cannot be seen or distinguished.
Reaction	No interest	Absence of response to the key elements of the experimental environment.
	Hiding behind owner	Positioning of the dog in order to use the owner as a barrier between him/herself and the other key elements of the experimental area.
	Hiding behind experimenter	Positioning of the dog in order to use the experimenter as a barrier between him/herself and the other key elements of the experimental area.
	Non-Codable	When the behaviour cannot be seen or distinguished.

* These two behaviours were further coded for the person in the room on whom the dogs jumped and sniffed (i.e., owner, experimenter, demonstrator A or demonstrator B).

## Data Availability

Data are available from the authors upon request.

## Supplementary Materials

The following supporting information can be downloaded at: https://www.mdpi.com/article/10.3390/ani13061027/s1, Table S1. Complete list of analysed subjects; Table S2. Table of Intraclass correlations between coders; Video S1. Example of a test trial: observation phase followed by response phase; Video S2. Example of the tail in the position raised-2, Video S3. Example of physical contact during sniffing.

## References

[1] Siniscalchi M., d’Ingeo S., Minunno M., Quaranta A. (2018). Communication in Dogs. Animals.

[2] Bloom T., Friedman H. (2013). Classifying dogs’ (*Canis familiaris*) facial expressions from photographs. Behav. Process..

[3] Waller B.M., Peirce K., Caeiro C.C., Scheider L., Burrows A.M., McCune S., Kaminski J. (2013). Paedomorphic facial expressions give dogs a selective advantage. PLoS ONE.

[4] Caeiro C., Guo K., Mills D. (2017). Dogs and humans respond to emotionally competent stimuli by producing different facial actions. Sci. Rep..

[5] Kaminski J., Hynds J., Morris P., Walker B.M. (2017). Human attention affects facial expressions in domestic dogs. Sci. Rep..

[6] Bekoff M. (1974). Social play and play-soliciting by infant canids. Am. Zool..

[7] Palagi E., Nicotra V., Cordoni G. (2015). Rapid mimicry and emotional contagion in domestic dogs. R. Soc. Open Sci..

[8] Quaranta A., Siniscalchi M., Vallortigara G. (2007). Asymmetric tail-wagging responses by dogs to different emotive stimuli. Curr. Biol..

[9] Feddersen-Petersen D.U. (2000). Vocalization of European wolves (*Canis lupus lupus* L.) and various dog breeds (*Canis lupus f. fam.)*. Arch. Anim. Breed..

[10] Pongrácz P., Molnár C., Miklósi Á. (2010). Barking in family dogs: An ethological approach. Vet. J..

[11] Siniscalchi M., Sasso R., Pepe A.M., Vallortigara G., Quaranta A. (2010). Dogs turn left to emotional stimuli. Behav. Brain Res..

[12] Nagasawa M., Murai K., Mogi K., Kikusui T. (2011). Dogs can discriminate human smiling faces from blank expressions. Anim. Cogn..

[13] Müller C.A., Schmitt K., Barber A.L., Huber L. (2015). Dogs can discriminate emotional expressions of human faces. Curr. Biol..

[14] Albuquerque N., Guo K., Wilkinson A., Savalli C., Otta E., Mills D. (2016). Dogs recognize dog and human emotions. Biol. Lett..

[15] Albuquerque N., Resende B. (2023). Dogs functionally respond to and use emotional information from human expressions. Evol. Hum. Sci..

[16] Albuquerque N., Guo K., Wilkinson A., Resende B., Mills D.S. (2018). Mouth-licking by dogs as a response to emotional stimuli. Behav. Process..

[17] Vas J., Topál J., Gácsi M., Miklósi A., Csányi V. (2005). A friend or an enemy? Dogs’ reaction to an unfamiliar person showing behavioural cues of threat and friendliness at different times. Appl. Anim. Behav. Sci..

[18] Albuquerque N., Mills D.S., Guo K., Wilkinson A., Resende B. (2021). Dogs can infer implicit information from human emotional expressions. Anim. Cogn..

[19] Perneger T.V. (1998). What’s wrong with Bonferroni adjustments. BMJ.

[20] Dror S., Sommese A., Miklósi Á., Temesi A., Fugazza C. (2022). Multisensory mental representation of objects in typical and Gifted Word Learner dogs. Anim. Cogn..

[21] Concha A., Mills D.S., Feugier A., Zulch H., Guest C., Harris R., Pike T.W. (2014). Using sniffing behavior to differentiate true negative from false negative responses in trained scent-detection dogs. Chem. Senses.

[22] Siniscalchi M., Sasso R., Pepe A.M., Dimatteo S., Vallortigara G., Quaranta A. (2011). Sniffing with the right nostril: Lateralization of response to odour stimuli by dogs. Anim. Behav..

[23] Jezierski T., Walczak M., Górecka A. Information-seeking behaviour of sniffer dogs during match-to-sample training in the scent lineup. Pol. Psychol. Bull..

[24] D’Aniello B., Semin G.R., Alterisio A., Aria M., Scandurra A. (2018). Interspecies transmission of emotional information via chemosignals: From humans to dogs (*Canis lupus familiaris*). Anim. Cogn..

[25] Siniscalchi M., d’Ingeo S., Fornelli S., Quaranta A. (2018). Lateralized behavior and cardiac activity of dogs in response to human emotional vocalizations. Sci. Rep..

[26] Koru E., Havlicek Z., Rezac P. (2018). Incidence of dogs jumping on household members upon entering their home in comparison with holding food. Appl. Anim. Behav. Sci..

[27] Rezac P., Koru E., Havlicek Z., Pospisilova D. (2017). Factors affecting dog jumping on people. Appl. Anim. Behav. Sci..

[28] Simon T., Guo K., Frasnelli E., Wilkinson A., Mills D.S. (2022). Testing of behavioural asymmetries as markers for brain lateralization of emotional states in pet dogs: A critical review. Neurosci. Biobehav. Rev..

[29] Racca A., Amadei E., Ligout S., Guo K., Meints K., Mills D. (2010). Discrimination of human and dog faces and inversion responses in domestic dogs (*Canis familiaris*). Anim. Cogn..

[30] Racca A., Guo K., Meints K., Mills D.S. (2012). Reading faces: Differential lateral gaze bias in processing canine and human facial expressions in dogs and 4-year-old children. PLoS ONE.

[31] Payne E., Bennett P.C., McGreevy P. (2015). Current perspectives on attachment and bonding in the dog–human dyad. Psychol. Res. Behav. Manag..

[32] Correia-Caeiro C., Guo K., Mills D. (2021). Bodily emotional expressions are a primary source of information for dogs, but not for humans. Anim. Cogn..

