# Supplementary material for: How Do Dogs Behave When Presented with Situations of Different Emotional Valences?
Source: Animals (Basel). 2023 Mar 11;13(6):1027. doi: 10.3390/ani13061027 (PMC10044040; doi:10.3390/ani13061027)
Supplement: Supplementary file 1 [file animals-13-01027-s001.zip › animals-2223655-supplementary.pdf]

Supplementary Material

How do dogs behave when presented with situations of different emotional valences?

*Paulo Souza[1], Kun Guo[2], Daniel S Mills[3], Briseida Resende[4], Natalia Albuquerque[4]**

[1]*Institute of Biosciences, University of São Paulo, Brazil*

[2]*School of Psychology, University of Lincoln, UK*

[3]*Department of Life Sciences, University of Lincoln, UK*

[4]*Institute of Psychology, University of São Paulo, Brazil*

correspondence to: nsalbuquerque@gmail.com

Table S1. complete list of analysed subjects

Dog	Breed	Age (Years)	Sex	Status
Caruso	Pug	6	Male	Neutered
Calabresa	Pug	4	Female	Neutered
Chérie	Stray	3	Female	Neutered
Preta	Stray	11	Female	Neutered
Maitê	Shetland Sheepdog	0.8	Female	Entire
Iara	Stray	2	Female	Neutered
Naná	Bernese	5	Female	Neutered
Willow	Bernese	7	Female	Neutered
Chico	Stray	2	Male	Neutered
Astana	Springer Spaniel	3	Female	Neutered
Duffy	Shih Tzu	1.6	Male	Neutered
Lunna	Stray	6.5	Female	Neutered
Juju	Stray	2	Female	Neutered
Penélope	Stray	2.5	Female	Neutered
Tora	Staffordshire Bull Terrier	1.5	Female	Neutered
Adele Valentina	Papillon	3.5	Female	Neutered
Zatar	Stray	4	Male	Neutered
Julie	Lhasa Apso Mix	2.5	Female	Neutered
Nix	Stray	5	Female	Neutered
Pudim	Poodle	3	Male	Neutered
Link	Stray	5	Male	Neutered
Colie	Stray	4	Female	Neutered
Lolla	Stray	3	Female	Neutered

Kevin	Shetland Sheepdog	4.1	Male	Neutered
Cacau	Stray	5	Female	Neutered
Bolota	Shih Tzu	2.1	Male	Entire
Thor	Golden Retriever	5	Male	Neutered
Chincha	Yorkshire	5	Male	Neutered
Estrelinha	Stray	2.5	Female	Neutered
Winnie	Stray	12	Female	Neutered
Liora Nina	Shetland Sheepdog	3.3	Female	Neutered
Spyke	Dachshund	5	Male	Entire
Nega	Stray	3.5	Female	Neutered
Luna	Lhasa Apso	3	Female	Entire
Milka	Shih Tzu	2.3	Female	Neutered
Bolota Bombolone	Pug	9	Male	Neutered
Polly	Stray	6	Female	Neutered
Guta	Schnauzer	5.5	Female	Neutered
Lua Catarina	Stray	3	Female	Neutered
Maria Quitéria	Giant Schnauzer	1.5	Female	Neutered
Layla	Golden Retriever	8	Female	Neutered
Capitu	Cocker Spaniel	5	Female	Neutered
Maria Quiteria	Stray	7	Female	Neutered
Miski	W. H. White Terrier	10	Female	Neutered
Melrose	W. H. White Terrier	10	Female	Neutered
ViviJr	W. H. White Terrier	12	Female	Neutered
Laika	Stray	3	Female	Neutered
Grappa	Labrador+Golden Mix	6	Female	Neutered
Google	Golden Retriever	7	Male	Neutered
Mambo	Golden Retriever	6	Male	Neutered
Luke Skywalker	Pug	3.7	Male	Neutered
Miky	Shih Tzu	6	Male	Neutered
Patrícia	Yorkshire	10	Female	Neutered
Tabata	Yorkshire	5	Female	Entire
Maria José	Yorkshire	5	Female	Entire
Maria Lúcia	Yorkshire	5	Female	Entire
Cacau	Basset Hound	1.9	Male	Entire
Luna	Basset Hound	3.9	Female	Neutered
Vitória	Stray	11	Female	Neutered
Penélope	Stray	6.8	Female	Neutered
Lord Zé	Pug	9	Male	Neutered
João	Yorkshire	7	Male	Neutered
Astato	Stray	5	Male	Neutered

Duquesa	Stray	3	Female	Neutered
Snoopy	Dachshund	3	Male	Neutered
Jobim	Jack Russel	3	Male	Entire
Lili	Poodle Mix	8.5	Female	Neutered
Toddy	Welsh Corgi	4	Male	Neutered
Simba	Stray	2	Male	Neutered

Table S2. Table of Intraclass correlations between coders

Behaviour	ICC	
	Intraclass correlation	p value
Neutral Posture	0,821	<0.001
Body towards Owner	0,942	<0.001
Body towards DemoA	0,968	<0.001
Body towards DemoB	0,936	<0.001
Body towards Table	0,984	<0.001
Body towards Bowl	0,953	<0.001
Body towards "Out"	0,957	<0.001
Head towards Owner	0,991	<0.001
Head towards Experimenter	0,99	<0.001
Head towards DemoA	0,978	<0.001
Head towards DemoB	0,996	<0.001
Head towards bowl	0,997	<0.001
Head towards "Out"	0,993	<0.001
Tail position Raised2	0,956	<0.001
Tail position Relaxed	0,8	<0.001
Tail movement Linear Horizontal	0,994	<0.001
Tail Moving	0,991	<0.001
Tail Not Moving	0,992	<0.001
Sniffing	0,993	<0.001
Jumping On	0,999	<0.001
Non Interest	0,991	<0.001

Video S1: Example of a test trial: observation phase followed by response phase

Video S2: Example of the tail in the position raised-2

Video S3: Example of physical contact during sniffing
